# The tip of the iceberg

**DOI:** 10.1002/jha2.117

**Published:** 2020-10-28

**Authors:** Rosario M. Morales‐Camacho, Almudena Aguilera‐Saborido, F. Javier Rodríguez‐Martorell, Ricardo Alcántara, Concepción Prats‐Martín, Emilio Franco‐Macías

**Affiliations:** ^1^ UGC de Hematología Universidad de Sevilla Sevilla Spain; ^2^ UGC de Cardiología y Cirugía Cardiovascular Universidad de Sevilla Sevilla Spain; ^3^ Departamento de Neurorradiología y Universidad de Sevilla Sevilla Spain; ^4^ Departamento de Neurología Hospital Universitario Virgen del Rocío, Instituto de Biomedicina de Sevilla (IBIS/CSIC/CIBERONC), Universidad de Sevilla Sevilla Spain

A 46‐year‐old man, previously healthy, was referred to a neurologist some days after his family witnessed an episode in which he suddenly was unable to answer a simple question while laughing for no apparent reason. In 2 minutes, he regained consciousness without recalling what had just happened. The neurological examination was normal. Auscultation revealed a grade I/VI mitral systodiastolic murmur. The laboratory workup revealed only a mildly prolonged activated partial thromboplastin time of 34.8 s (normal 20‐30). A brain magnetic resonance imaging detected an old left frontal infarct (left). An electroencephalogram showed bitemporal epileptiform abnormalities. Supra‐aortic duplex and transcranial Doppler examination were normal. A transthoracic echocardiogram demonstrated 10 and 9 mm vegetations on the atrial side of the mitral valve (top right, green, and blue fine lines), and mild mitral regurgitation by color Doppler (bottom right). Finally, testing for thrombophilia, lupus anticoagulant (LAC) (Russell test), anticardiolipin (aCL) IgG 80.9 GPL/mL (reference range 0‐20), and anti‐β2 glycoprotein I (aβ2‐GPI) Ig G 126.6 (normal 0‐20) were positive. No other autoimmune diseases or infectious causes were found. The antiphospholipid antibodies were again positive 3 months later.

**FIGURE 1:**
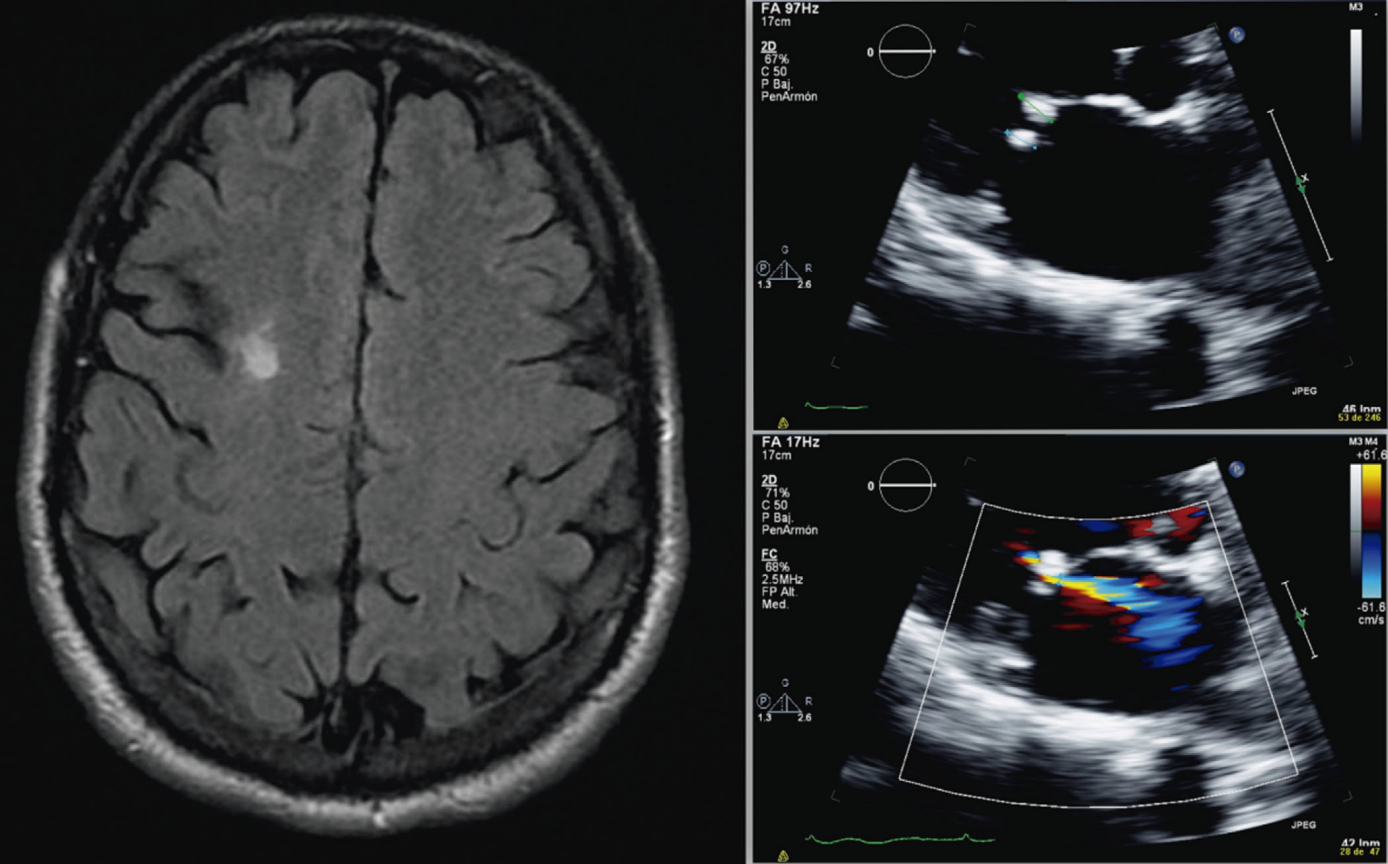


A focal seizure was the clinical onset of a primary antiphospholipid syndrome (APS). An uncertain‐to‐date stroke and Libman‐Sacks endocarditis were unveiled. The patient was started on levetiracetam 500 mg each 12 hours and anticoagulation with acenocumarol. One year after his initial diagnosis, he has had neither new neurological symptoms nor thrombotic events.

Seizures, strokes, and Libman‐Sacks endocarditis are associated with APS. Triple positivity for antiphospholipid antibodies (LAC, aCL, and aβ2‐GPI) shows the strongest association with thrombotic events. This case demonstrates that a minor paroxysmal event may be just the tip of the iceberg of a severe autoimmune thrombotic disease.

